# Serum metabolomics reveal the mechanisms by which fermented brewer’s spent grains promote intestinal development in white-feathered broilers

**DOI:** 10.3389/fvets.2025.1614917

**Published:** 2025-10-10

**Authors:** Yuanfeng Li, Zhiheng Meng, Yiyuan Wang

**Affiliations:** College of Agriculture and Biology, Liaocheng University, Liaocheng, China

**Keywords:** fermented brewer’s spent grains, intestinal development, broilers, metabolomics, metabolic mechanism

## Abstract

This study aimed to investigate the effects of wet-fermented brewer’s grains (WFBGs) on gut development and serum metabolism in white-feathered broilers. A total of 192 one-day-old male broilers (initial body weight: 36.46 ± 0.93 g) were randomly assigned to two treatment groups: the control group (0% WFBGs) and the experimental group (20% WFBG inclusion), with 6 replicates of 16 birds per replicate. The results of intestinal morphological parameters, quantified using ImageJ software after hematoxylin–eosin (HE) staining, showed that compared with the control group, broilers fed a diet supplemented with 20% WFBGs had significantly improved duodenal development. Specifically, the duodenal villus height (VH) increased by 10.2% (*p* < 0.05), and the villus height-to-crypt depth ratio (VH/CD) increased by 27.2% (*p* < 0.05)—both indicators reflecting enhanced duodenal development. Through untargeted metabolomics analysis for screening differentially expressed metabolites (DEMs) from serum samples, 211 DEMs were identified, including 98 upregulated DEMs and 113 downregulated DEMs in the WFBG group. KEGG pathway enrichment analysis revealed that these DEMs were significantly associated with key metabolic processes, including linoleic metabolic pathways, linoleic acid metabolism, phenylalanine metabolism, and other relevant pathways. Specifically, key DEMs involved in amino acid metabolism included significantly increased phenylalanine levels and decreased 4-HPA and 3-HPA levels (*p* < 0.05). In conclusion, the inclusion of 20% WFBGs in the diet of white-feathered broilers significantly promoted intestinal development. These favorable outcomes are tied to modified serum metabolic profiles and shifts in lipid and amino acid metabolism in broilers, underscoring WFBG’s significance for boosting broiler intestinal development while also adding to the theoretical framework for poultry by-product-based feeds.

## 1 Introduction

Against the backdrop of escalating geopolitical conflicts, the global food crisis is becoming increasingly severe, and it is extremely urgent to find and develop alternative animal feed materials. China has a huge production of brewer’s grains, which are often directly discharged as waste, resulting in serious resource waste (1). It is essential to effectively apply brewer’s grains to livestock and poultry production. Brewer’s grains are rich in amino acids, trace elements, and proteins, making them a high-quality feed material for animal production (2). Previous studies have shown that dried brewer’s grains (DBGs) have been used in poultry (3–5) and livestock (6), helping to modulate the gut microbiota balance (7) and increase the apparent digestibility of nutrients (4), thus improving growth and reducing breeding costs (8). Wet brewer’s grains (WBGs) have also been used in livestock and poultry feed (9). Due to their unique digestive and metabolic properties in the rumen, WBGs are more commonly used in ruminants (10) than in poultry (7). Fermentation technology can effectively preserve and improve the nutritional content of brewer’s grains, enhancing their value as animal feed (1). After fermentation, the apparent digestibility of nutrients from DBGs was significantly increased in laying hens (9) or ducks (3). Previous studies have demonstrated that probiotics and fermentation metabolites derived from fermented wheat bran enhance intestinal barrier function (11), regulate gut microbiota homeostasis (12), and alleviate intestinal inflammation (13). Other studies on broilers show common fibrous additives are typically included at 2–12% of the basal diet (5), and ≥15% is generally considered “high-dose” for their altering feed intake, nutrient digestibility, or intestinal physiology (14, 15). However, there is currently a lack of research on the application of high-dose wet-fermented brewer’s grains (WFBGs) to broiler chickens.

Metabolomics is commonly used as a technique for biomarker discovery, which can analyze metabolites in biological fluids, cells, and tissues (16). Abnormal changes in small molecule metabolites within the body are usually the ultimate response of the organism to the effect of disease or stress (17). Metabolomics of stress-induced biological samples may identify diagnostic biomarkers and reveal stress mechanisms. Clarifying these mechanisms through the investigation of intestinal development and serum metabolism may provide a bridge for studies related to the regulation of WFBGs (18). Serum metabolites can characterize the body’s metabolism and inflammatory response (18). Serum metabolomics can better demonstrate the correlation between intestinal development and organs and pathways (19). Serum metabolomics proves useful for capturing digestive efficiency, intestinal structural features, and dynamic diet-linked metabolic changes in broilers and other livestock, thus serving as a more holistic and application-friendly sample type to assess how WFBGs in diets impact intestinal development (20). In recent years, metabolomics technology has been widely used to evaluate fermented feed in pigs (21), ducks (22), and broilers (23). To the best of our knowledge, there are no reports investigating how metabolomics can identify serum metabolites regulating intestinal development in WFBG-fed broilers.

In broiler production, the intestinal development of broiler chickens directly determines their growth rate in the later stages. Previous studies have found that fermented feed or fiber raw materials can promote intestinal development through regulating gut microbiota (24, 25), but whether high-dose addition causes damage to intestinal development has not been reported. Therefore, it is hypothesized that the addition of WFBGs may promote the intestinal development of white-feathered broilers, and thus, this study intends to explore the impacts of WFBGs on their intestinal development and serum metabolism.

## 2 Materials and methods

### 2.1 Animals and treatments

A total of 192 one-day-old (36.5 ± 0.93 g) white-feathered broilers (hybrid of Cobb breed cock and Hy-line brown laying hen) were randomly divided into two groups (6 replicates of 16 birds each). The control and experimental groups were fed a basic diet containing 0% WFBGs or 20% WFBGs (basic feed to WFBG ratio of 80:20), respectively. This trial lasted for 21 days. All birds were housed in wire cages (70 cm × 80 cm × 40 cm). The birds were purchased from Aoxiang Poultry Industry Co., Ltd., Liaocheng, Shandong Province, China. During this study, all birds had free access to feed and water. The basic feed for birds was purchased from a commercial feed enterprise (Haiding Feed Co., Ltd., Liaocheng, China). The WFBGs used in this experiment were prepared in the laboratory of Liaocheng University (Liaocheng, Shandong, China) (WBG: corn: wheat bran: fermenting agent = 45:23:30:2, incubated at 37°C for 72 h, once a week). This study was approved by the Animal Protocol Review Committee of Liaocheng University, China (No: LCU20240016). The fermenting agent (composed of *Lactobacillus acidophilus*, *Bifidobacterium* spp., and *Bacillus subtilis*) was purchased from Qingdao Genyuan Biotechnology Group (Qingdao, Shandong Province, China). The contents of crude protein and crude fiber of fermented WFBGs increased from 9.22 to 10.23% and decreased from 8.40 to 7.20%, respectively.

### 2.2 Sample collection

On day 21, one bird (fasted for 12 h, with body weight close to the average of each replicate) was randomly selected from each replicate of the control and 20% WFBG groups for sample collection. Briefly, 2–3 mL of blood samples were collected from broilers’ wing veins using a sterile syringe, then left to stand at room temperature for 2 h for natural coagulation. Subsequently, serum samples were separated by centrifugation at 5,000 *g* for 10 min (4°C) and then stored at −80°C for metabolomics analysis. The birds were euthanized with exsanguination under sodium pentobarbital anesthesia (60 mg·kg^−1^) after blood collection. Under strict aseptic procedures, the middle segments of the duodenum, jejunum, and ileum (each approximately 2 cm in length) were carefully removed and fixed in 4% formalin for subsequent intestinal morphological observations.

### 2.3 Intestinal morphology

After being dehydrated and embedded in paraffin, the fixed intestinal tissues (5 μm thickness) were continuously sliced and stained with hematoxylin–eosin (26). The intestinal mucosal structure was observed using a microscope and analyzed using Media Cybernetics imaging software (Image-Pro Plus 6.0, USA). The villus height (VH) and crypt depth (CD) of 16 intact villi per slice were measured, and 5 slices were selected to calculate the average value of each tissue.

### 2.4 Serum metabolite extraction

#### 2.4.1 Serum preparation for metabolomics

Before analysis, serum samples were thawed on ice and mixed thoroughly by vortexing for 10 s. A volume of 100 μL of serum was mixed with 400 μL of extraction solution (MeOH: ACN, 1:1 (v/v), containing deuterated internal standards). The mixture was vortexed for 30 s, sonicated for 10 min in a 4°C water bath, and incubated at −40°C for 1 h to precipitate proteins. Samples were then centrifuged at 12000 rpm for 15 min at 4°C. The supernatant was transferred to a fresh glass vial and injected into the ultra-high liquid chromatography–tandem mass spectrometry (UHPLC–MS/MS) system for analysis. An equal aliquot of the supernatant of all samples was fixed to prepare the quality control (QC) sample.

#### 2.4.2 Metabolomics data capture

For positive and negative metabolites, LC–MS/MS analyses were performed using a UHPLC system (Orbitrap Exploris, Thermo Fisher Scientific, USA), coupled with UPLC BEH Amide (Waters ACQUITY, 2.1 mm × 50 mm, 1.7 μm) and MS (Orbitrap Exploris 120, Thermo Fisher Scientific, USA). The mobile phase consisted of solvent A (water containing 25 mmol/L of ammonium acetate and 25 mmol/L of ammonia hydroxide, pH = 9.75) and solvent B (ACN, 0.1% formic acid). Other parameters were as follows: autosampler temperature, 4°C; autosampler mode, partial loop injection; sample loop volume, 10 μL; injection volume, 2 μL. The MS (Orbitrap Exploris 120, Thermo Fisher Scientific, USA) was used to evaluate the full scan MS spectrum in information-dependent acquisition mode, controlled by the Xcalibur (V4.4) software (Thermo Fisher Scientific, USA). Further details regarding metabolomics analysis are provided in Supplementary file S1.

### 2.5 Metabolomics data analysis

The raw data were converted to the mzXML format using ProteoWizard software (ProteoWizard Software Foundation, USA) and processed with an in-house program (developed using R and based on XCMS). The R package and BiotreeDB (V3.0, China) were applied to metabolite annotation (27). Candidate metabolites (VIP > 1 and adjusted *p* < 0.05) were regarded as potential biomarkers.

### 2.6 Statistical analysis

Data on intestinal morphology were analyzed using Student’s *t*-test in SPSS software (V23.0; SPSS Inc., Chicago, Illinois, USA) (28), with the pen considered as a fixed effect. All data are expressed as means. *p*-values < 0.05 were considered statistically significant.

## 3 Results

### 3.1 Intestinal morphology

Compared to the control group, the duodenum VH and VH/CD ratio in the 20% WFBG group were significantly increased (*p* < 0.05, Table 1). No differences were observed in CD in the duodenum, VH, CD, or VH/CD ratio in the jejunum or ileum (*p* > 0.05, Table 1).

**Table 1:** Effects of dietary WFBGs on the intestinal development in broiler chickens.^1^

Items^2^	CON group^3^	20% WFBG group^3^	SEM^4^	*P*-value
Duodenum				
VH, mm	1.27^b^	1.40^a^	0.057	0.045
CD, mm	0.24	0.20	0.017	0.052
VH/CD	5.48^b^	6.97^a^	0.524	0.018
Jejunum				
VH, mm	0.65	0.76	0.079	0.208
CD, mm	0.15	0.15	0.021	0.884
VH/CD	4.58	5.17	0.575	0.331
Ileum				
VH, mm	0.37	0.36	0.032	0.908
CD, mm	0.11	0.11	0.011	0.782
VH/CD	3.47	3.41	0.505	0.908

^1^Means represent 6 replicates with 16 birds per cage (n = 6/group).^2^VH, villus height; CD, crypt depth. ^3^CON group: basal diet; 20% WFBG group: basal diet + 20% WFBG. ^4^Pooled standard error of mean. Values within a row with different superscripts differ significantly at ^a^*P* < 0.05.

### 3.2 Serum untargeted metabolomics profile of WFBGs

#### 3.2.1 Quality control of metabolite data from serum samples

**Figure 1:**
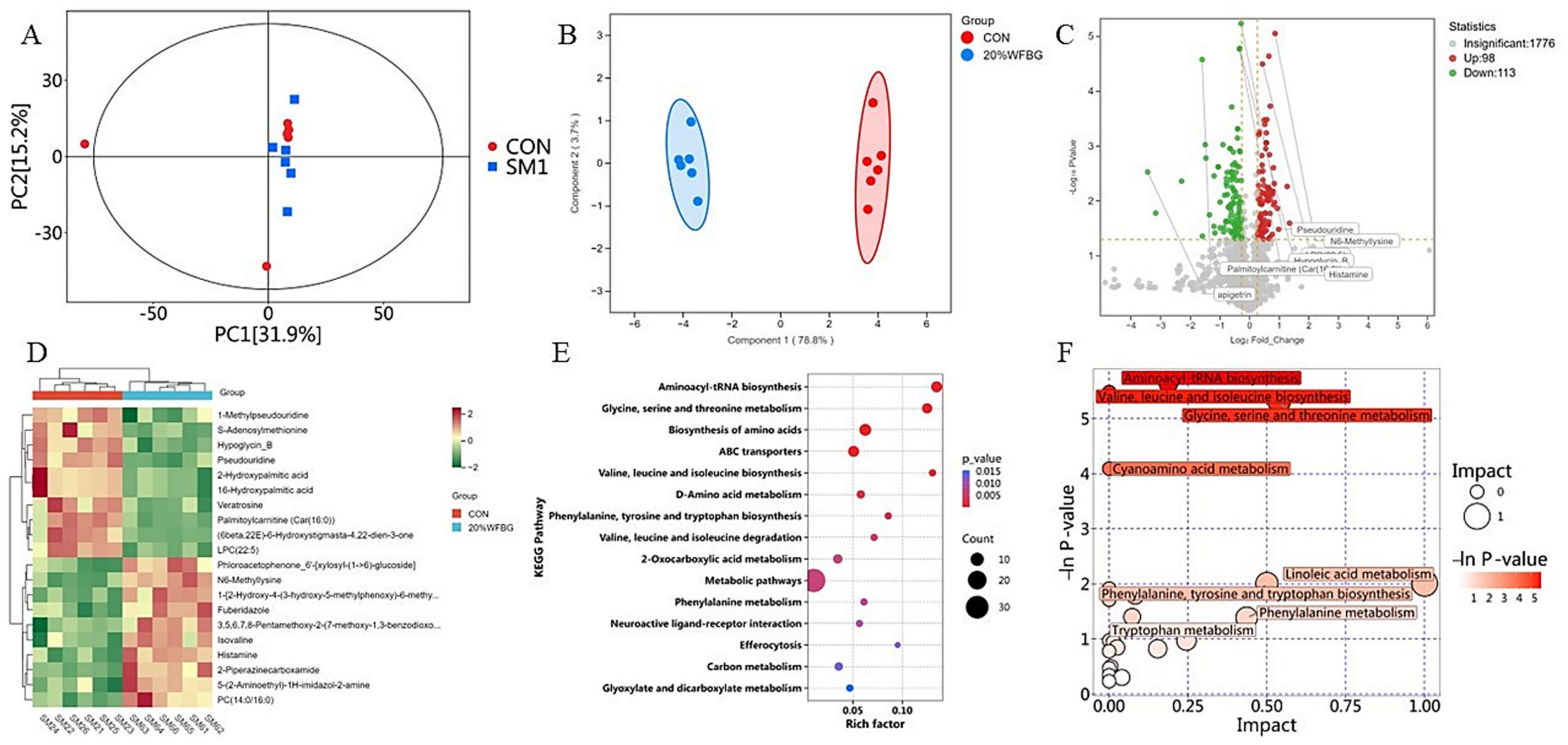
Serum metabolome analysis in broilers with WFBGs and normal birds. **(A)** PCA-DA plots of serum metabolites in the positive and negative ion modes. **(B)** OPLS-DA analysis of alterations in the metabolite profile. **(C)** Volcano plot of DEMs between the control and 20% WFBG groups (*p*-value of *t*-test < 0.05 and fold change (FC) > 1.2, or FC < 0.833). Gray, no change; red, upregulation; green, downregulation. **(D)** Cluster heat map of DEMs: from green (less) to red (more). **(E)** The enrichment map of metabolic pathways of DEMs from the control and 20% WFBG group comparisons. X-axis, rich factor; y-axis, enriched pathway; size of the bubble, the number of DEMs enriched in the pathway; color, enrichment significance. CON, control group; SM1, 20% WFBG group. **(F)** The analysis diagram of metabolic pathways. Each bubble is a metabolic pathway; the horizontal axis and size of the bubble represent the size of the influencing factor of the pathway in topological analysis. The vertical axis and the color of the bubble represent the negative natural logarithm of the *p*-value of enrichment analysis.

In this study, pooled QC samples were used to validate the system performance. These tightly clustered QC samples demonstrated that the metabolomic method had good repeatability and stability (Figure 1A). Serum samples of broiler chickens revealed significant changes in serum metabolic profiles of broiler chickens fed 20% WFBG diets (Figure 1A). OPLS-DA score plots (Figure 1B) revealed distinct clustering patterns between the control and WFBG groups, demonstrating the model’s robustness with parameters: R^2^X = 0.332, R^2^Y = 0.991, and Q^2^ = 0.477, indicating no overfitting.

#### 3.2.2 Differential metabolite analysis

The screening criteria used to identify differently expressed metabolites (DEMs) in the serum of broiler chickens from the 20% WFBG and control groups were VIP score > 1, fold change (FC) > 1.2, or FC < 0.833, and *p*-value < 0.05. There were 211 DEMs in the serum (Figure 1C), including 98 upregulated DEMs and 113 downregulated DEMs in the 20% WFBG group compared to the control group. Among these DEMs, dehydroalanine and N6-mmethyllysine, related to amino acid metabolism, were upregulated, while benzamide was downregulated. In lipid metabolism and energy regulation, propionylcarnitine [Car(3:0)], 2-hydroxy-1-[hydroxy(7-methoxy-2-oxo-2H-1-benzopyran-6-yl)methyl]-2-methylpropyl ester, 3-methylbutanoic acid, and 2-[(1-methyl-1H-pyrazolo[3,4-d]pyrimidin-4-yl)amino]ethanol were upregulated, whereas palmitoylcarnitine [Car(16:0)], linoleoyl carnitine [Car(18:2)], oleoylcarnitine [Car(18:1)], and 5*α*-pregnan-3α,17-diol-20-one 3-sulfate were downregulated. For antioxidant and inflammatory regulation, taxifolin and kaempferol 3-α-L-arabinoside were upregulated, while formoterol and apigetrin were downregulated. In drug metabolism and xenobiotic handling, bezafibrate and 2-chloro-5-nitro-N-phenyl compound were upregulated, and 5-fluoro-1,3-dihydro-2H-benzimidazole-2-thione was downregulated. Tricin methyl ether (6β,22E)-6-hydroxystigmasta-4,22-dien-3-one, (3E)-4-(2-carboxyphenyl)-2-oxobut-3-enoate, and benzamide, related to hormone signaling and nitrogen metabolism, were downregulated, while N-acetyl-D-galactosaminitol, associated with carbohydrate metabolism and glycoconjugates, was upregulated. Hierarchical clustering analysis according to the relative abundance of serum metabolites was conducted to generate a heatmap (Figure 1D). The top 10 DEMs in the 20% WFBG and control groups are shown in Table 2. More detailed results are shown in Supplementary Table 1.

**Table 2:** Identification results of top-10 DEMs in the serum between the 20% WFBG and control groups.

No.	Metabolites	m/z	RT(s)	VIP	FC	Mode	*P*-value
1	Kaempferol 3-.alpha.-L-arabinoside	419.1	169.8	1.33	1.79	up	0.0106
2	Butanoic acid, 3-methyl-, 2-hydroxy-1-[hydroxy(7-methoxy-2-oxo-2H-1-benzopyran-6-yl)methyl]-2-methylpropyl ester	379.18	128.6	2.1	1.78	up	0.0048
3	Bezafibrate	362.11	112	1.99	1.71	up	0.0067
4	Taxifolin	303.05	116.8	1.2	2.4	up	0.0054
5	Benzamide, 2-chloro-5-nitro-N-phenyl-	275.02	96.6	1.9	1.97	up	0.0327
6	N-Acetyl-D-galactosaminitol	224.11	146.1	2.11	1.74	up	0.0025
7	Propionylcarnitine [Car(3:0)]	218.14	171.1	2.06	1.91	up	0.0135
8	2-[(1-Methyl-1H-pyrazolo[3,4-d]pyrimidin-4-yl)amino]ethanol	194.1	32.2	2.01	1.75	up	0.0118
9	N6-Methyllysine	161.13	288.7	2.52	1.81	up	0
10	Dehydroalanine	88.04	231.8	1.82	2.54	up	0.0252
11	Apigetrin	431.1	131.7	2.56	0.09	down	0.0029
12	(6beta,22E)-6-Hydroxystigmasta-4,22-dien-3-one	427.36	90	2.6	0.35	down	0.0009
13	Car(18:1)	426.36	90	2.56	0.36	down	0.0016
14	Linoleoylcarnitine [Car(18:2)]	424.34	93.4	2.41	0.2	down	0.0043
15	5.alpha.-Pregnan-3.alpha.,17-diol-20-one 3-sulfate	413.2	159.5	2.06	0.44	down	0.0035
16	Palmitoylcarnitine [Car(16:0)]	400.34	95.4	2.57	0.33	down	0
17	Formoterol	345.18	275.5	1.96	0.43	down	0.0289
18	Tricin methyl ether	343.09	25.3	1.98	0.39	down	0.0178
19	(3E)-4-(2-Carboxyphenyl)-2-oxobut-3-enoate	238.07	99.4	1.91	0.33	down	0.0435
20	5-Fluoro-1,3-dihydro-2H-benzimidazole-2-thione	167.01	14	1.56	0.11	down	0.0165

WFBGs, wet fermented brewer’s grains; m/z, mass-to-charge ratio; RT, retention time; FC, fold change, 20% WFBG group vs. control group; VIP, variable importance in the projection; DEMs, differentially expressed metabolites.

#### 3.2.3 KEGG pathway analysis

A total of 1987 metabolites in the positive and negative ion modes were submitted to the KEGG pathway analysis, of which 211 were DEMs (Supplementary Table 1). Key functional impacts of enriched DEMs on metabolic pathways were determined by the MetaboAnalyst (V 6.0) (Figure 1E). The enriched DEMs between the 20% WFBG and control groups had a significant effect on 51 metabolic pathways, such as metabolic pathways, biosynthesis of amino acids, ABC transporters, 2-oxocarboxylic acid metabolism, biosynthesis of cofactors, carbon metabolism, linoleic acid (LA) metabolism, and phenylalanine metabolism (*p* < 0.05). There were 32 metabolites involved in metabolic pathways, 8 metabolites involved in the biosynthesis of amino acids, 7 metabolites involved in ABC transporters, 5 metabolites involved in 2-oxocarboxylic acid metabolism, 5 metabolites in biosynthesis of cofactors, 4 metabolites in carbon metabolism, 1 metabolite involved in LA metabolism, and 3 metabolites involved in phenylalanine metabolism. Notably, linoleic acid (LA) metabolism and phenylalanine metabolism exhibited pathway impact values of 1.000 and 0.436 (Figure 1F), respectively. These two pathways are likely to exert pivotal effects on mediating how WFBGs regulate intestinal development in broilers. Specifically, LA metabolism serves as a core biosynthetic pathway for polyunsaturated fatty acids such as arachidonic acid. These lipid metabolites are capable of enhancing the structural integrity of intestinal epithelial tight junctions and mitigating intestinal inflammatory reactions by inhibiting the activation of the nuclear factor-κB (NF-κB) signaling pathway, ultimately reinforcing the protective capacity of the intestinal barrier. In parallel, phenylalanine metabolism is intricately involved in the biosynthesis of two distinct classes of bioactive molecules: neurotransmitters (e.g., dopamine, which regulates gastrointestinal motility and mucosal nerve signaling) and antioxidants (e.g., melanin, a potent scavenger of reactive oxygen species (ROS) in mucosal tissues). For broilers supplemented with WFBGs, this dual biosynthetic role of phenylalanine metabolism could potentially alleviate oxidative stress in the intestinal mucosa and fine-tune the balance of intestinal mucosal immunity—a mechanism that ultimately supports the maintenance of intestinal homeostasis. Therefore, we further investigated the changes in LA and phenylalanine metabolites in the serum of broiler chickens fed 20% WFBG diets. The detailed results of metabolic pathway analysis are shown in Supplementary Table 2.

#### 3.2.4 LA and phenylalanine targeted metabolomic studies

**Figure 2:**
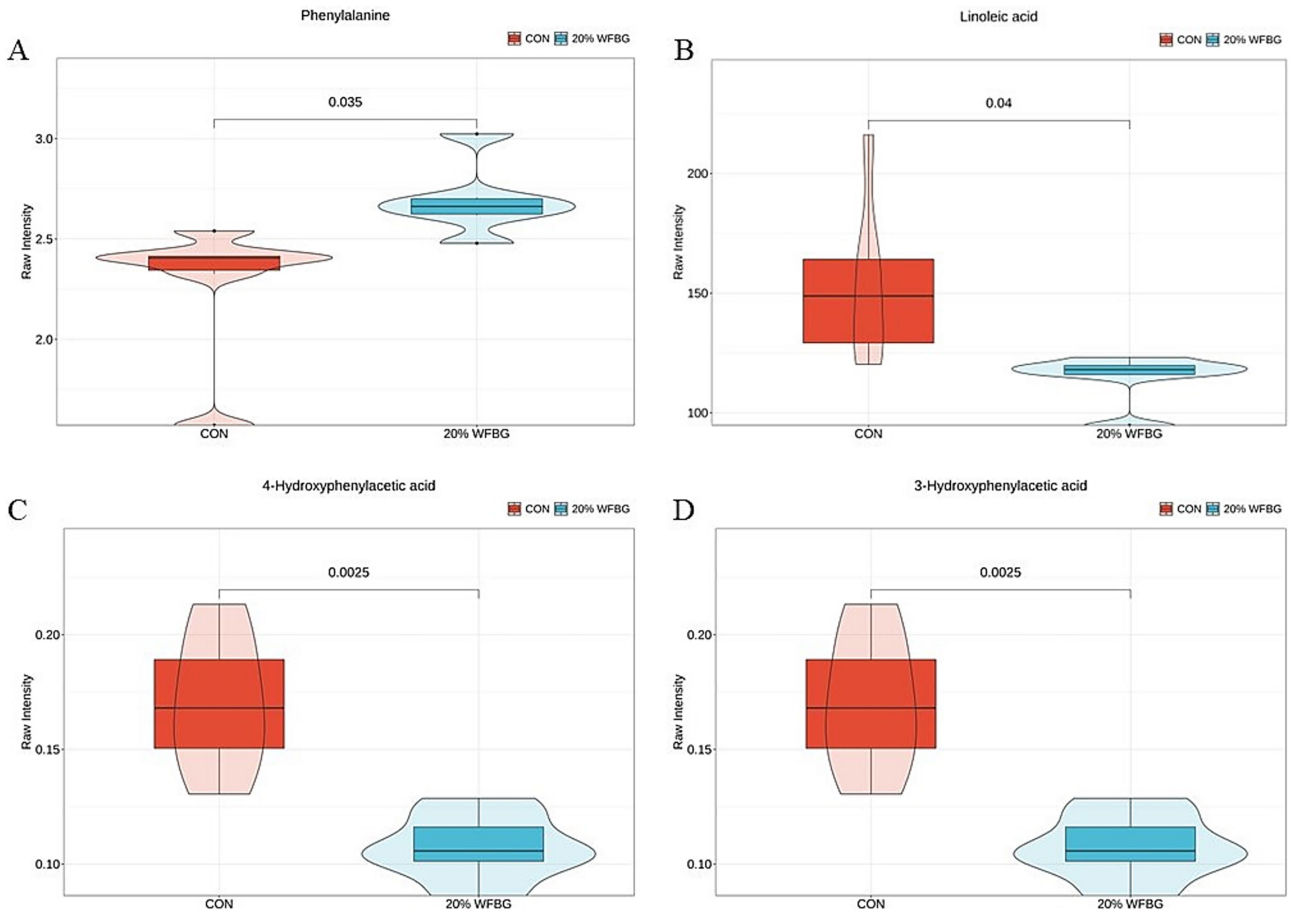
Concentrations of serum metabolites involved in LA metabolism and phenylalanine metabolism by targeted analysis. LA, linoleic acid. *p < 0.05, compared with the control group. CON, control group. 20% WFBG, basic feed to WFBG ratio of 80:20.

The quantitative analysis results of phenylalanine metabolites and LA metabolites are presented in Figures 2A,B, respectively. Specifically, in the 20% WFBG group, the level of phenylalanine was significantly increased (*p* < 0.05, Figure 2A), the levels of LA (*p* < 0.05, Figure 2B), 4-hydroxyphenylacetic acid (*p* < 0.05, Figure 2C), and 3-hydroxyphenylacetic acid (*p* < 0.05, Figure 2D) were significantly decreased.

## 4 Discussion

### 4.1 Intestinal development

The quality of intestinal development greatly affects animal growth. The small intestine is a crucial digestive and absorptive organ in the body, and an appropriate amount of fiber can alter the villous morphology of the small intestine. The changes in villus morphology can be reflected in the variations in VH and CD (27). Higher VH and CD values indicate a larger absorption area and higher cell proliferation activity, respectively. The VH/CD ratio is commonly used for the comprehensive evaluation of small intestine digestive capacity. An increase in intestinal length will increase food retention time in the intestine, thereby increasing the absorption of nutrients. The increase in CD can enhance cell proliferation activity, while the decrease in CD may reflect a slower rate of cell generation. Previous studies demonstrated that a 2–10% dried BG diet did not promote intestinal development in broilers (29), and a 2–12% DBG diet had no effect on the VH, CD, or VH/CD in slow-growing broiler chickens (5). Our previous study demonstrated that the jejunum VH and VH/CD ratio in pullets fed with a 20% WFBG diet was significantly increased (unpublished data). In this study, compared with the control group, the duodenal VH and VH/CD ratio in the 20% WFBG group were significantly increased (*p* < 0.05, Table 1), but no significant differences were observed in the jejunum and ileum (*p* > 0.05, Table 1). The duodenum (the primary site for the initial digestion of carbohydrates, proteins, and lipids) exhibits heightened sensitivity to dietary stimuli. This heightened responsiveness directly underlies the notable morphological alterations observed in the duodenum, distinguishing it from other intestinal segments. In contrast, the jejunum (the main site for absorbing amino acids, fatty acids, and vitamins) receives chyme that has already undergone partial digestion in the duodenum. This prior digestion step reduces the intensity of WFBG-associated stimuli reaching the jejunum, thereby weakening potential morphological effects. In parallel, the ileum functions as a post-absorption segment, characterized by a well-developed mucosal barrier and specialized immune cell populations. These features endow the ileum with a more robust functional buffering capacity to counteract external dietary perturbations. Given these functional differences across intestinal segments, neither the jejunum nor the ileum showed detectable significant morphological changes—a result that aligns with observations from prior studies focusing on dietary fiber-induced intestinal adaptations in broiler chickens (30). WFBG metabolites modulate duodenal pH to facilitate villus development. They may weakly adjust jejunal digestive enzyme activity and ileal microbiota. These observations are hypothesized to stem from the appropriate fiber content in WFBGs, as the fiber promotes the development of duodenal mucosal structures while avoiding imposing an excessive metabolic burden on the broiler.

### 4.2 Serum untargeted metabolomics profile of WFBGs

With the application of the WFBG diet in broilers, an untargeted UHPLC–MS/MS metabolomics study of serum was performed to investigate the growth-promoting mechanism of WFBGs. To investigate the growth-promoting mechanism of WFBGs in broilers, we performed an untargeted metabolomic profiling using UHPLC–MS/MS to analyze the serum metabolome of broilers fed the WFBG diet. A total of 211 DEMs were significantly observed in the WFBG group. These DEMs not only quantify WFBG’s regulatory effect on broiler metabolism but also provide a key “metabolic signature” for decoding its growth-promoting mechanisms. Biologically, this metabolomic variation indicates that the WFBG drives systematic remodeling of the broiler serum metabolome, likely through its bioactive components (e.g., fiber and polyphenols) interacting with broilers’ digestion, absorption, and hepatic metabolism processes. Downregulated DEMs may reduce metabolic burden and inflammation, while upregulated DEMs may enhance protein synthesis—a process critical for broiler growth (31). Notably, these DEMs establish a link between WFBG intake and broiler growth phenotypes.

Specifically, dehydroalanine—upregulated in the WFBG group—supports intestinal barrier repair and protein digestion, while N6-methyllysine (also upregulated) enhances functional protein activity; both address the gut’s growth-related demands. Downregulated benzamide, by contrast, lowers the accumulation of toxic ammonia, thereby protecting the intestinal mucosa. For energy metabolism and gut health: upregulated propionylcarnitine boosts intestinal ATP supply, 3-methylbutanoic acid inhibits pathogens, and flavonoid ester reduces lipid oxidation. On the other hand, downregulated long-chain acylcarnitines [Car(16:0), Car(18:2), Car(18:1)—common subtypes in broilers] and steroid metabolites ease oxidative stress and excessive inflammation. In terms of oxidative stress regulation, upregulated taxifolin and kaempferol 3-*α*-L-arabinoside activate the Nrf2/HO-1 pathway to clear ROS, which helps improve the duodenal VH/CD ratio. Downregulated formoterol and apigetrin, meanwhile, prevent unwanted functional disruptions. Other key changes: upregulated bezafibrate and 2-chloro-5-nitro-N-phenyl compound strengthen xenobiotic clearance; downregulated 5-fluoro-1,3-dihydro-2H-benzimidazole-2-thione reduces potential toxicity. Additionally, downregulated tricin methyl ether and (6β,22E)-6-hydroxystigmasta-4,22-dien-3-one maintain hormonal and nitrogen balance, while upregulated N-acetyl-D-galactosaminitol supports glycoconjugate synthesis to sustain intestinal function. Pathway analysis showed that these metabolites were mainly involved in metabolic pathways, biosynthesis of amino acids, ABC transporters, 2-oxocarboxylic acid metabolism, biosynthesis of cofactors, carbon metabolism, LA metabolism, and phenylalanine metabolism. Changes in these serum metabolites and the altered metabolic pathways may provide new evidence to understand the action pathway of WFBGs.

Lipids, the main components of cell membranes, directly maintain the physiological functions of cells and participate in the transport of triglycerides (32). Growing evidence has shown that animal growth is associated with the alteration of lipid metabolism (33). LA, n-6 polyunsaturated fatty acids, plays an important role in inhibiting fatty acid synthesis by regulating the expression of enzymes or receptors related to fatty acid synthesis, thereby reducing fat production (34, 35). LA also promotes mitochondrial biogenesis and alleviates acute lung injury (36) and has shown pro-tumorigenic (37) or anti-tumorigenic effects in multiple types of cancer (38). LA metabolism is an important metabolic pathway involving many important physiological functions, including the oxidation of fatty acids to LA, activating glucose and lipid metabolism processes to maintain blood glucose stability and promote cellular metabolic activity. The most important function of LA metabolism is the control of blood sugar. LA metabolism can promote the oxidation of saturated fatty acids, thereby reducing the production of cholesterol and triglycerides, and ultimately lowering blood lipid levels. Furthermore, LA also strengthened the intestinal epithelial barrier by regulating the NF-κB/MLCK pathway in mice (39). It was reported that conjugated LA regulated gut microbiota-host metabolic and immunomodulatory interactions (40). In this study, it was found that LA metabolism was disturbed in broiler chickens fed with 20% FBG, with a decrease in LA metabolites. Other metabolites closely related to LA metabolism, such as arachidonic acid, gamma-linolenic acid, 13(S)-HODE, and 9-HPODE, were also detected, but no significant changes were observed. These findings indicated that high doses of WFBGs may have a negative impact on lipid metabolism changes in broiler chickens. More animal experiments are needed for further verification.

In addition to LA metabolites, the changed metabolites (phenylalanine, 4-hydroxyphenylacetic acid, 4-HPA, and 3-hydroxyphenylacetic acid, 3-HPA) in this study are also involved in the phenylalanine metabolism pathway. Phenylalanine is an important essential amino acid. Phenylalanine metabolism deficiency can lead to various diseases. Phenylalanine in the plasma is mainly metabolized and converted into tyrosine in the body, which is closely related to the formation of certain hormones and neurotransmitters. If phenylalanine metabolism is disrupted, the conversion of phenylalanine to tyrosine is blocked. Increased phenylalanine concentration and decreased tyrosine concentration in the blood, or an increase in the phenylalanine/tyrosine ratio, can cause various diseases, especially damage to the nervous system. Qin et al. (38) reported that excessive phenylalanine reduced glucose utilization in the liver, exacerbated pancreatic fat deposition, and induced pancreatic injury and glucose metabolism disorders in growing and fattening pigs (38). It has been reported that remodeling gut microbiota and phenylalanine metabolism can effectively inhibit pulmonary inflammatory damage induced by *Mycoplasma gallisepticum* in chickens (41). 4-HPA, a major microbiota-derived metabolite of polyphenols, participates in antioxidant activity. 4-HPA might regulate immune function and improve intestinal health in meat pigeons (42). 3-HPA, formed by gut microbiota, might increase the release of nitric oxide from the endothelial layer, causing vasodilation and ultimately lowering blood pressure in the body (43). In this study, increased phenylalanine and decreased 4-HPA and 3-HPA levels were observed in the serum from the 20% WFBG group, which might indicate that phenylalanine metabolism plays a significant role in the growth and development of broiler chickens. Elevated phenylalanine levels may supply substrates for intestinal development by promoting amino acid transport and protein synthesis in intestinal epithelial cells (44). Reduced 4-HPA and 3-HPA—microbiota-derived metabolites—may alleviate inhibition of the mTOR pathway, activating villus cell proliferation signals (45). Collectively, these may synergistically promote intestinal morphological development by enhancing amino acid utilization and regulating the cell cycle, though experimental validation of metabolic pathway bidirectional regulation is warranted.

With the application of a UHPLC–MS/MS-based metabolomics approach, 211 serum DEMs involved in metabolic pathways, biosynthesis of amino acids, ABC transporters, 2-oxocarboxylic acid metabolism, biosynthesis of cofactors, carbon metabolism, LA metabolism, and phenylalanine metabolism were identified, which indicated that WFBGs might affect amino acid metabolism and lipid metabolism by regulating changes in GM. These DEMs may be potential biomarkers for WFBGs promoting the growth of broiler chickens. However, the mechanistic exploration of how WFBGs link to metabolic and intestinal changes remains incomplete, preventing the establishment of a causal chain between WFBG intake, gut microbial shifts, and downstream metabolic/intestinal responses. Additionally, the long-term effects and potential safety risks of WFBGs were not evaluated, precluding assessment of their long-term impacts on broiler health or product quality.

## 5 Conclusion

This study shows that adding 20% WFBGs to broiler diets effectively promotes intestinal development. Metabolomic analysis reveals that WFBGs modulate LA and phenylalanine metabolism, boosting nutrient absorption and energy balance—laying a metabolic foundation to clarify its gut health benefits. Sourced from brewery waste, WFBGs are an economical feed ingredient fitting circular economy principles. The 20% inclusion offers a scientific reference for feed formulation, cutting reliance on conventional feeds and supporting brewery waste’s industrial use in animal nutrition.

## Data Availability

The metabolic data generated in this study have been deposited in Metabolights under accession number MTBLS13078. The data are publicly available at https://www.ebi.ac.uk/metabolights/MTBLS13078.
