# Supplementary material for: Serum metabolomics reveal the mechanisms by which fermented brewer’s spent grains promote intestinal development in white-feathered broilers
Source: Front Vet Sci. 2025 Oct 10;12:1614917. doi: 10.3389/fvets.2025.1614917 (PMC12551227; doi:10.3389/fvets.2025.1614917)
Supplement: Supplementary file 1 [file Data_Sheet_1.ZIP › Supplement files/Supplementary file S1.docx]

**Metabolomics data acquisition**

The gradient elution conditions of LC were as follows with a flow rate of 0.3 ml min−1: 10%–25% solvent B for 0–4 min; 25%–55% solvent B for 4–7 min; 55%–100% solvent B for 7–28 min; 100 solvent B for 28–29 min; and 100%–10% solvent B for 29–30 min. The column temperature was maintained at 30 °C.

The ESI source conditions of MS were set as following: sheath gas flow rate, 50 Arb; Aux gas flow rate, 15 Arb; capillary temperature, 320 ℃; full MS resolution, 60000; MS/MS resolution, 15000; collision energy, SNCE 20/30/40; spray voltage, 3.8 kV (positive) or -3.4 kV (negative), respectively.

**Multivariate statistical analysis**

The raw data were converted to the mzXML format using ProteoWizard and processed with an in-house program. which was developed using R and based on XCMS, for feature detection, extraction, alignment, and integration. The R package and the BiotreeDB (V3.0) were applied in metabolite identification.

In this study, X features were detected and X metabolites were left after relative standard deviation de-noising. Then, the missing values were filled up by half of the minimum value. Also, internal standard normalization method was employed in this data analysis. The final dataset containing the information of feature number, sample name and normalized feature area was imported to SIMCA18.0.1 software package (Sartorius Stedim Data Analytics AB, Umea, Sweden) for multivariate analysis. Data was scaled and logarithmic transformed to minimize the impact of both noise and high variance of the variables. After these transformations, PCA (principle component analysis, PCA), an unsupervised analysis that reduces the dimension of the data, was carried out to visualize the distribution and the grouping of the samples. 95% confidence interval in the PCA score plot was used as the threshold to identify potential outliers in the dataset.

In order to visualize group separation and find significantly changed metabolites, supervised orthogonal projections to latent structures discriminate analysis (OPLS-DA) was applied. Then, a 7-fold cross validation was performed to calculate the value of R2 and Q2. R2 indicates how well the variation of a variable is explained and Q2 means how well a variable could be predicted. To check the robustness and predictive ability of the OPLS-DA model, a 200 times permutations was further conducted. Afterward, the R2 and Q2 intercept values were obtained. Here, the intercept value of Q2 represents the robustness of the model, the risk of overfitting and the reliability of the model, which will be the smaller the better.

Furthermore, the value of variable importance in the projection (VIP) of the first principal component in OPLS-DA analysis was obtained. It summarizes the contribution of each variable to the model. The metabolites with VIP>1 and *P* < 0.05 (student t test) were considered as significantly changed metabolites. In addition, commercial databased including KEGG (http://www.genome.jp/kegg/) and MetaboAnalyst (http://www.metaboanalyst.ca/) were used for pathway enrichment analysis.
